# Sublethal RNA Oxidation as a Mechanism for Neurodegenerative Disease

**DOI:** 10.3390/ijms9050789

**Published:** 2008-05-20

**Authors:** Rudy J. Castellani, Akihiko Nunomura, Raj K. Rolston, Paula I. Moreira, Atsushi Takeda, George Perry, Mark A. Smith

**Affiliations:** 1 Department of Pathology, University of Maryland, Baltimore, Maryland, USA; 2 Department of Neuropsychiatry, Interdisciplinary Graduate School of Medicine and Engineering, University of Yamanashi, Yamanashi, Japan; 3 Department of Pathology, Case Western Reserve University, Cleveland, Ohio, USA; 4 Center for Neuroscience and Cell Biology of Coimbra, University of Coimbra, Coimbra, Portugal; 5 Department of Neurology, Tohoku University School of Medicine, Sendai, Japan; 6 College of Sciences, University of Texas at San Antonio, San Antonio, Texas, USA

**Keywords:** Alzheimer disease, 8-oxoguanosine, neurodegeneration, oxidative damage, Parkinson disease, RNA

## Abstract

Although cellular RNA is subjected to the same oxidative insults as DNA and other cellular macromolecules, oxidative damage to RNA has not been a major focus in investigations of the biological consequences of free radical damage. In fact, because it is largely single-stranded and its bases lack the protection of hydrogen bonding and binding by specific proteins, RNA may be more susceptible to oxidative insults than is DNA. Oxidative damage to protein-coding RNA or non-coding RNA will, in turn, potentially cause errors in proteins and/or dysregulation of gene expression. While less lethal than mutations in the genome, such sublethal insults to cells might be associated with underlying mechanisms of several chronic diseases, including neurodegenerative disease. Recently, oxidative RNA damage has been described in several neurodegenerative diseases including Alzheimer disease, Parkinson disease, dementia with Lewy bodies, and prion diseases. Of particular interest, oxidative RNA damage can be demonstrated in vulnerable neurons early in disease, suggesting that RNA oxidation may actively contribute to the onset of the disease. An increasing body of evidence suggests that, mechanistically speaking, the detrimental effects of oxidative RNA damage to protein synthesis are attenuated, at least in part, by the existence of protective mechanisms that prevent the incorporation of the damaged ribonucleotides into the translational machinery. Further investigations aimed at understanding the processing mechanisms related to oxidative RNA damage and its consequences may provide significant insights into the pathogenesis of neurodegenerative and other degenerative diseases and lead to better therapeutic strategies.

## 1. Introduction

Neurodegenerative diseases are common and are strictly age-associated; for example, the prevalence in the United States per 1,000 elderly is 65 for Alzheimer disease (AD) and 9.5 for Parkinson disease (PD), while the annual incidence per 1,000,000 of general population is 1.6 for amyotrophic lateral sclerosis (ALS) [1]. Many lines of evidence indicate that oxidative damage is involved in the pathogenesis of neurodegenerative diseases including AD, PD, and ALS [2–9]. Indeed, oxidatively modified products of nucleic acids (e.g., 8-oxodeoxyguanosine, 8-oxoguanosine) and proteins (e.g., 3-nitrotyrosine, protein carbonyls), as well as products of lipid peroxidation (e.g., 4-hydroxynonenal, F2-isoprostane, malondialdehyde) and glycoxidation (e.g., carboxymethyl-lysine, pentosidine), all known markers of oxidative damage, have been demonstrated in central nervous system lesions, and in ante-mortem cerebrospinal fluid, serum, and urine from patients with these diseases [2–9]. The increased levels of oxidative damage in such neurodegenerative diseases are often accompanied by the concomitantly reduced levels of anti-oxidative defense mechanisms in the same subjects [3, 5]. Remarkably, a number of genetic and environmental factors, namely disease-specific gene mutations, risk-modifying gene polymorphisms, and risk-modifying life-style factors are closely associated with oxidative damage [3, 6, 9], yet interventions such as the administration of one or several antioxidants have been, at best, only modestly successful in clinical trials. The complexity of reactive oxygen species (ROS) metabolism thus suggests that the interventions to date have been too simplistic. Instead, more integrated approaches may be required to not only enrich the exogenous antioxidants but also to up-regulate the endogenous anti-oxidative defense systems [8, 9]. Clearly, there is a considerable need for a better understanding of the association between ROS metabolism and neurodegeneration, particularly with respect to RNA oxidation, and whether amelioration of RNA oxidation comprises an effective avenue to treatment, experimentally and clinically.

Although RNA is subject to the same oxidative insults as DNA and other cellular macromolecules, oxidative damage to RNA has not been a major focus in oxidative stress studies. This is somewhat surprising since RNA is largely single-stranded and, its bases not being protected by hydrogen bonding or binding proteins, it is, in theory, more susceptible to oxidative insults than DNA [10–13]. It might also be noted that the relative abundance of RNA and its subcellular distribution in the immediate vicinity of mitochondria suggest an additional level of RNA vulnerability to ROS [10]. Given these factors, it is not surprising that, using high-performance liquid chromatography coupled with electrochemical detector (HPLC-ECD) or with electrospray tandem mass spectrometry (HPLC-MS/MS), greater oxidation of RNA than of DNA has been shown [14] in both cell lines and tissues, including human leukocytes [15], human skin fibroblasts [16], human lung epithelial cells [17] and in rat liver [14, 18]. Moreover, urinary excretion of an oxidized form of ribonucleoside in healthy humans and rats [19] not only suggests substantial RNA oxidation in normal metabolism but also the existence of a repair mechanism for the damaged RNA.

It is now evident that RNA molecules are not only intermediates for the transfer of genetic information but also key players in many mechanisms controlling the expression of genetic information [20–23]. Given that our understanding of RNAs is undergoing a “renaissance,” we and others have developed a hypothesis that RNA damage is involved in the pathogenesis of neurodegenerative diseases [10–12, 21–23]. Here we review recent studies demonstrating RNA oxidation in several neurological diseases and discuss the biological significance of such damage to RNA, as well as possible cellular mechanisms of repair against this damage.

## 2. Significance of RNA Studies in Neurodegenerative Disease Research

Recent progress in genetics has revealed an expansion of the role of RNA beyond its classical function in the “Central Dogma.” It is now evident that only a minority of genetic transcripts (2–3% in human) code for proteins. Non-coding RNA (ncRNA), rather than being cast aside as “junk,” functions directly in structural and catalytic activities and also plays a critical role in regulating the timing and rate of gene expression [20–23]. Of particular note, the complexity of an organism correlates poorly with the number of protein coding genes; however, complexity is highly correlated with the number of ncRNAs [24]. Furthermore, the increasing variety of ncRNAs being identified in the CNS suggests a strong connection between the biogenesis, dynamics of action, and combinational regulatory potential of ncRNAs and the complexity of the CNS [22, 23]. Therefore, further advances in studies on the mechanisms and consequences of RNA damage and its surveillance may have a significant impact on our understanding of the pathophysiology of currently unresolved complex diseases including neurological and psychiatric diseases [22–25].

In familial forms of two of the most common neurodegenerative diseases, AD and PD [1], germline mutations cause familial autosomal dominant disease with specific protein aggregates (e.g., amyloid-β in AD and α-synuclein in PD) that form hallmark lesions in affected brains, suggesting a possible etiological role of the protein aggregates in disease [8, 26]. However, the vast majority of the patients with AD and PD have no known germline mutations, prompting investigations into other upstream events. As we have reviewed here, oxidative damage to neuronal RNA is not only a common feature of AD, PD, and associated neurodegenerative diseases, but is also an early event, suggesting involvement of RNA damage as a primary pathogenic mechanism. Interestingly, RNA damage is less lethal for cells than mutations in genomic DNA, raising the novel possibility of sublethal RNA oxidation as an underlying mechanism of chronic disease and, in particular, neurodegenerative disease.

## 3. RNA Oxidation in Various Neurological Diseases

The disruption of transcriptional or translational fidelity in neurons leads to the accumulation of aberrant or misfolded proteins and neuronal death [27, 28]. Oxidative damage to DNA has been well studied and several classes of products such as base oxidation and fragmentation products (e.g., single- and double-strand breaks), inter/intra-strand cross-links, DNA-protein cross-links, and sugar fragmentation products have been identified [29, 30]. However, few studies have focused on oxidative damage to RNA and only limited kinds of oxidatively modified bases in RNA have been reported previously [31–36]. Among multiple adducts of nucleoside oxidation, adducts of deoxyguanosine and guanosine, i.e., 8-oxodeoxyguanosine (8-OdG) and 8-oxoguanosine (8-OG) are two of the best characterized and studied forms of DNA and RNA oxidation, respectively [14–18].

The availability of specific antibodies to 8-OdG and 8-OG has enabled us to perform *in situ* examination of nucleoside oxidation in postmortem brain tissue [37, 38]. In 1999, increased levels of 8-OdG/8-OG were demonstrated in the vulnerable neuronal populations in postmortem brains of patients with AD and PD [10, 39]. In AD and PD, the neuronal 8-OdG/8-OG showed cytoplasmic predominance, which suggested either mitochondrial DNA or cytoplasmic RNA as major targets of oxidative damage. Because the neuronal 8-OdG/8-OG immunoreactivities in AD brain were diminished greatly by RNase pretreatment but not by DNase pretreatment, we concluded that the oxidized nucleoside was predominantly associated with RNA rather than DNA [10]. This idea was further supported by the immunoelectron microscopic observation that most of the oxidized nucleoside was localized to ribosomes [40].

Similar RNA oxidation was also observed in brain samples of patients with Down syndrome [41], dementia with Lewy bodies [42], Creutzfeldt-Jakob disease [43], and subacute sclerosing panencephalitis [44]. The oxidative damage to RNA was demonstrated not only in sporadic-forms of the diseases but also in familial-forms of AD [45] and prion diseases, e.g., familial Creutzfeldt-Jakob disease and Gerstmann-Strausler-Scheinker disease [43, 46]. Moreover, nuclear DNA oxidation and cytoplasmic RNA oxidation were observed in brains of patients with a genetic defect of nucleotide excision repair, *xeroderma pigmentosum*, a condition that manifests clinically in its hypersensitivity to sunlight and progressive neurological disease [47]. Additionally, RNA oxidation was demonstrated in muscle cells of patients with rimmed vacuole myopathy [48], a neuromuscular disease characterized by accumulation of proteins associated with neurodegenerative disease. RNA oxidation was also seen in smooth muscle and endothelial cells of atherosclerotic plaques [49], which are a known risk for AD pathology. In aged human skeletal muscle, a recent study has also demonstrated increased RNA oxidation, possibly related to increased levels of non-heme iron [50]. These findings further the concept that RNA oxidation is involved in chronic neurodegeneration.

Our immunocytochemical studies of neuronal RNA oxidation were followed by biochemical detection of the oxidized nucleoside in AD brain with immunoblot analysis [51–55]. Shan *et al*. [51, 55] used northwestern blotting, a method for detecting oxidized RNA using specific antibody, in this case, monoclonal anti-8-OG antibody, and showed that a significant amount of brain poly (A)+ mRNA species were oxidized in AD. The oxidation of mRNA was confirmed by cDNA synthesis and Southern blotting of the immunoprecipitated mRNA species. Densitometric analysis of the Southern blot results revealed that 30–70% of the mRNAs from AD frontal cortices were oxidized, while only 2% of the mRNAs were oxidized in age-matched controls [55]. Interestingly, reverse transcription polymerase chain reaction (RT-PCR) and filter array analyses of the identified oxidized mRNAs revealed that, while some species were more susceptible to oxidative damage in AD, no common motifs or structures were found in the oxidatively susceptible mRNA species. Some of the identified known oxidized transcripts were related to AD, which included p21ras, mitogen-activated protein kinase (MAPK) kinase 1, carbonyl reductase, copper/zinc superoxide dismutase (SOD1), apolipoprotein D, calpains, but not amyloid-β protein precursor or tau [51]. Although these studies by Shan *et al*. [51, 55] focused on mRNA species, Honda *et al*. [52] and Ding *et al*. [53, 54] reported that ribosomal RNA (rRNA), extremely abundant in neurons, contained 8-OG in AD brain. Remarkably, rRNA showed higher binding capacity to redox-active iron than transfer RNA (tRNA), and consequently the oxidation of rRNA by the Fenton reaction formed 13 times more 8-OG than that formed with tRNA [52].

Of note, both immunocytochemical [9, 10, 39, 42] and biochemical [51, 53] studies revealed that the regional distribution of RNA oxidation in the brain correlated with the selective neuronal vulnerability in each neurological disease. There were increased levels of 8-OG in the hippocampus and cerebral neocortex in AD and in the substantia nigra in PD, while no alteration in 8-OG levels was found in the cerebellum in either AD or PD compared with controls [9, 10, 39, 51, 53]. Immunocytochemical analysis further indicated that the oxidized RNAs were more abundant in neuronal cells compared with glial cells [10, 39–42, 45].

In addition to the brain, significantly increased levels of the oxidized RNA nucleoside, 8-OG, have been identified in cerebrospinal fluid from patients with AD and PD [56–58] as well as in serum of PD patients [57], suggesting 8-OG as a possible biomarker for these diseases. As we describe in the following section, 8-OG may have diagnostic utility as a marker of early stage disease.

## 4. Experimental Models

Experimental studies in rodents have shown that neuronal RNA oxidation (8-OG) and spatial memory deficit are observed in older animals [59] as well as animals with intermittent hypoxia [60]. In both the aging and the hypoxia models, antioxidants or mitochondrial metabolites can reduce oxidative damage and the spatial memory deficit. C57BL/6J mice, including young mice (10–12 weeks old), show substantially increased levels of spontaneously oxidized RNA (8-OG) in neurons of the hippocampus and the substantia nigra [61], which contrasts with human control brains that show no apparent level of RNA oxidation at younger ages [10]. It would be interesting to see whether RNA oxidation differs among other mammalian species, particularly at the outer limit of species lifespan, since this time point tends to *negatively* correlate with levels of DNA oxidation [62].

Animal models of neurodegeneration via neurotoxins demonstrate RNA oxidation. Animals treated with 1-methyl-4-phenyl-1,2,3,6-tetrahydropyridine (MPTP), for example, show degeneration in nigrostriatal dopaminergic neurons [63] and a significant increase in neuronal 8-OG in the substantia nigra [61]. In addition, a kainic acid-mediated excitotoxic model for neurodegeneration [64] is associated with increased levels of 8-OG in hippocampal neurons and glial cells [65].

A strong genetic link between oxidative damage and neurodegeneration has been suggested by the finding that about 20% of patients with familial ALS carry a mutation in SOD1, a metalloenzyme that catalyzes the dismutation of the toxic superoxide (O_2_•^−^) to hydrogen peroxide (H_2_O_2_) [66]. Although the prevailing hypothesis in SOD1 ALS suggests a toxic gain of function [5], a transgenic mouse model of ALS expressing Gly93Ala-SOD1 mutation [67] shows increased RNA oxidation in the motor neurons of the spinal cord [55, 68].

Cell culture experiments further suggest an association between increased RNA oxidation and neurodegeneration [69, 70]. In a mixed astrocyte and neuron culture model [69], DNA and RNA oxidation have been observed following proteasome inhibition, a biochemical abnormality commonly observed in neurodegenerative disease. Interestingly, in this model, neurons demonstrated larger increases in nucleic acid oxidation compared to astrocytes, and RNA appeared to undergo a greater degree of oxidation than DNA, a finding similar to the AD brain [10].

Various neurodegenerative diseases including AD, PD, and ALS are associated with defects in the ubiquitin-proteasome system which has been shown to affect multiple aspects of RNA metabolism [71]. Another recent study using primary rat cortical cultures has shown that exposures to oxidative stress causes neuronal RNA oxidation and subsequent neuronal death [70], suggesting again an upstream role of neuronal RNA oxidation in the process of neurodegeneration.

## 5. Time Course of RNA Oxidation

The involvement of RNA oxidation in a variety of neurological diseases raises the possibility that this mechanism is epiphenomenal. In this respect, it should be noted that RNA oxidation is seen in early-stage AD [9, 40] as well as in a presymptomatic case with a familial AD mutation [45], Down syndrome cases with early-stage AD pathology [41], and subjects with mild cognitive impairment (MCI) [53, 54]. Moreover, the increased level of RNA oxidation in cerebrospinal fluid (CSF) is more prominent in AD and PD of short duration [56, 58] as well as in AD patients with higher scores on cognitive testing [56]. Recent studies of MCI subjects have also demonstrated increased oxidation/nitration to protein and lipid peroxidation [72, 73], increased lipid peroxidation in CSF, plasma, and urine [74], increased DNA oxidation in peripheral leukocytes [75], decreased plasma antioxidant vitamins and enzymes [76], and decreased plasma total antioxidant capacity [77]. In ALS, neuronal RNA oxidation has not been reported; however, significantly increased RNA oxidation has been observed in motor neurons in the presymptomatic stage in Gly93 Ala-SOD1 mice, the transgenic animal model of familial ALS [55, 68].

Of note, neuronal RNA oxidation is seen not only in early stage neurodegeneration but also in cases with subacute sclerosing panencephalitis, a condition caused by persistent measles virus infection and accompanied by neurofibrillary tangles [44]. From the standpoint of therapeutic intervention, early involvement of oxidative damage in disease pathogenesis provides a strategic target, as we have previously reviewed [9, 78, 79].

The relatively early chronological appearance of neuronal RNA oxidation is further suggested by a primary rat cortical culture model [70]. In the time course after various oxidative insults to the cultures, RNA oxidation occurs primarily in a distinct group of neurons that survive the insult but subsequently die in a delayed fashion. This finding models our hypothesis that RNA oxidation results in sublethal injury, predisposing to chronic neurodegeneration [70].

While protein carbonyls, lipid peroxidation products and glycoxidation products are relatively stable due to the formation of cross-links, oxidized RNAs are likely turned over more rapidly. On the other hand, RNA oxidation reflects the “steady-state balance” of oxidative damage at a “snapshot” point [10, 80]. In accordance with this concept, protein carbonyls, lipid peroxidation products such as 4-hydroxynonenal and F2-isoprostane, and a glycoxidation product carboxymethyl-lysine have been demonstrated in neurons with and without associated pathology [81–84]. These data likely reflect the occurrence of damage throughout the early- and advanced-stages of neurodegeneration. These observations contrast remarkably with RNA oxidation, a “steady-state” marker that is prominent in neurons without pathology and is present in lesser amounts in neurons containing pathology [40, 41].

3-Nitrotyrosine may be another steady-state marker of oxidative damage. 3-Nitrotyrosine is formed by a modification of tyrosine residue of proteins by an attack of peroxynitrite (ONOO−), a powerful oxidant produced from the reaction of O_2_•^−^ with nitric oxide (NO•), and is not known to accumulate in cells. As such, it is not surprising that intracellular level of 3-nitrotyrosine parallels the level of 8-OG in AD and Down syndrome brains [40, 41].

## 6. Types of Reactive Oxygen Species and Relevance to RNA

The brain is especially vulnerable to oxidative damage because of its high content of unsaturated fatty acids, high oxygen consumption rate and relative paucity of antioxidant enzymes compared with other organs [85, 86]. Given this environment, neurons are continuously exposed to ROS such as O_2_•^−^, H_2_O_2_, and hydroxyl radical (•OH) that are produced by cellular respiration [85–87]. •OH, on the other hand, can diffuse through tissue only in the order of several nanometers [88] and O_2_•^−^ is minimally permeable through cell membranes [89]. Generally speaking, it is thought that cytoplasmic RNA is a major target of •OH while highly diffusible H_2_O_2_ [90] reacts with redox-active metals through the Fenton reaction [52] to damage DNA.

In the AD brain, disrupted mitochondria likely also play a central role in producing abundant ROS and supplying redox-active iron into the cytosol [8, 91–93]. Indeed, ribosomes purified from AD hippocampus contain significantly higher levels of redox-active iron compared to controls, and the iron, in turn, is bound to rRNA [52]. Mitochondrial abnormalities coupled with metal dysregulation of metal homeostasis may therefore be key features closely associated with RNA oxidation in AD [94]. Interestingly, mitochondrial abnormalities [95, 96] and metal ion dysregulation [97, 98] are also found in the substantia nigra of PD, suggesting a common theme in neurodegenerative disease.

## 7. Pathogenic Cascade Initiated by RNA Oxidation

More than 20 different types of oxidatively altered purine and pyrimidine bases have been detected in nucleic acids [29, 30, 36, 99]. However, since guanine is the most reactive of the nucleic acid bases [33], it is not surprising that 8-hydroxyguanine is the most abundant [12]. The 8-hydroxyguanine-containing nucleoside, 8-OG, can be formed in RNA by direct oxidation of the base and also by the incorporation of the oxidized base from the cytosolic pool into RNA through the normal action of RNA polymerase [33, 99]. 8-OG, as well as 8-hydroxyadenosine, 5-hydroxycytidine, and 5-hydroxyuridine, have been identified in oxidized RNA [33], which may alter the pairing capacity and thus comprise the biochemical basis for erroneous translation, with 8-hydroxyguanine pairing with both adenine and cytosine [99, 100].

The biological consequence of oxidatively damaged mRNA species has been investigated *in vitro* by expressing oxidized mRNA species in cell lines. Oxidized mRNAs lead to loss of protein level and function, and potentially produce defective proteins leading to protein aggregation [51]. In a recent study, polyribosome analysis indicates that oxidized bases in mRNAs cause ribosome “stalling”, which leads to a decrease of protein expression [70]. When oxidized and non-oxidized luciferase RNAs were subjected to translation in rabbit reticulocyte lysates and analyzed by northern blot, the oxidized RNA samples showed decreased free monosomes and increased RNA-associated polyribosomes compared to the non-oxidized RNA samples [70]. In another recent study, the translation of oxidized mRNA in cell lines caused accumulation of short polypeptides, due to premature termination of translation of the oxidixed mRNA and/or proteolytic degradation of the modified protein containing the translation errors [101]. Coincidently, oxidative damage to Escherichia coli 16S rRNA results in the formation of short cDNA by the RT-PCR [102]. The biological consequences of ribosomal oxidation have been investigated *in vitro* using translation assays with oxidized ribosomes from rabbit reticulocytes and which demonstrate a significant reduction of protein synthesis [52]. Notably, studies on brains of subjects with AD and MCI have demonstrated ribosomal dysfunction associated with oxidative RNA damage [53, 54]. Isolated polyribosome complexes from AD and MCI brains further show a decreased rate of and capability for protein synthesis without alteration in the polyribosome content. Decreased rRNA and tRNA levels and increased 8-OG in total RNA pool, especially in rRNA, are accompanied by ribosomal dysfunction, while there is no alteration in the level of initiation factors [53]. These findings indicate that RNA oxidation has detrimental effects regardless of whether the damaged RNA species are coding for proteins (mRNA) or performing translation (rRNA and tRNA).

It is noteworthy in this respect that studies on some anti-cancer agents have shown that RNA damage can lead to cell-cycle arrest and cell death, via a p53-dependent mechanism associated with inhibition of protein synthesis or p53-independent mechanism as yet uncharacterized [103].

## 8. Repair Mechanisms

Degradation of RNA plays a central role in cellular metabolism and damaged RNA can be removed through degradation by ribonucleases (RNase), but selective degradation of oxidized RNA has not been established for known RNases [11, 104]. Oxidative stress induces cytoplasmic mRNA processing bodies (so called P-bodies), the site of active degradation of mRNA [105, 106], which is coupled to induction of another cytoplasmic structure called the “stress granules” [107]. In contrast to mRNAs with rapid turnover, stable RNAs, consisting primarily of rRNAs and tRNAs and encompassing 98% of total cellular RNA, may be protected against RNase action by a protective tertiary structure, assembly into ribonucleoprotein complexes, or even blocking the RNA's 3' terminus [104].

Until relatively recently, damaged RNA was thought to only be degraded and not repaired. However, Aas *et al*. [108] have suggested that cells have at least one specific mechanism to repair RNA damage [12, 103, 109]. Indeed, alkylation damage in RNA is repaired by the same mechanism as a DNA-repair, catalyzed in the bacterium Escerichia coli by the enzyme AlkB, and in humans by the related protein [108]. Alk B and its homologues hABH3 and hABH2 cause hydroxylation of the methyl group on damaged DNA and RNA bases, and thus directly reverse alkylation damage. Alk B and hABH3, but not hABH2, repair RNA, since Alk B and hABH3 prefer single-stranded nucleic acids while hABH2 acts more efficiently on double-stranded DNA [108]. DNA damage can be repaired not only by the mechanism of direct reversal of the modified bases but also by a base excision repair mechanism. Specific DNA glycosylases excise the damaged base and DNA polymerases replace the nucleotide [103, 110]. However, because the excision repair generally requires a complementary strand, the mechanism is not likely efficient in RNA [109].

Cells have mechanisms of dealing with nucleotide damage other than direct excision and repair, which seems to be useful for defense against oxidative damage to both DNA and RNA. Because oxidation of nucleotides can occur in the cellular nucleotide pool, and oxidized nucleotides can potentially be incorporated into DNA and RNA, the mechanism preventing incorporation of the oxidized nucleotide comes into play in coping with nucleic acid damage [11, 12, 103]. MutT protein in Escerichia coli and its mammalian homologues MutT homologue 1 (MTH1) and Nudix type 5 (NUDT5) proteins participate in this error-avoiding mechanism by hydrolyzing the oxidized nucleoside diphosphates and /or triphosphates to the monophosphates [99, 100, 110–113]. Indeed, the increase in the production of erroneous proteins by oxidative damage is 28-fold over the wild type cells in Escerichia coli mutT deficient cells, which is reduced to 1.2- or 1.4-fold by the expression of MTH1 or NUDT5, respectively [99]. Correspondingly, MTH1 deficiency leads to increased levels of RNA oxidation products induced by kainic acid treatment in the MTH1-null mouse [65]. An increased expression of human MTH1 in the vulnerable neuronal populations has been demonstrated in postmortem brains of AD [114] and PD [115], which may indicate a compensatory up-regulation of the MTH1 against oxidative stress [112].

In addition to the hydrolyzing action of MTH1 and NUDT5, several other enzymes involved in nucleotide metabolism show discriminatory activity against the oxidized nucleotides. Guanylate kinase (GK), an enzyme that converts GMP to GDP, is inactive on 8-OH-GMP. Similarly, ribonucleotide reductase (RNR), an enzyme that catalyzes reduction of four naturally occurring ribonucleoside diphosphates, is inactive in converting 8-OH-GDP to 8-OH-dGDP thus preventing incorporation of the oxidized nucleotide into DNA synthesis [111]. On the other hand, nucleotide diphosphate kinase (NDK), an enzyme that converts GDP to GTP, fails to show such discriminating function [111]. The final “gatekeeper” discriminating the oxidized nucleotide from normal nucleotide is RNA polymerase that incorporates 8-OH-GTP into RNA at a much lower rate compared to normal GTP incorporation [11, 100].

One important unanswered question is whether cells have machinery to deal with oxidatively damaged nucleotides that are contained in RNA, since RNA can be directly oxidized even if the incorporation of oxidized nucleotides into RNA is blocked. Recently, proteins that bind specifically to 8-OG-containing RNA have been reported, namely, Escerichia coli polynucleotide phosphorylase (Pnp) protein and human PNP [116, 117] as well as human Y box-binding protein 1 (YB-1) [118]. The binding of the specific protein likely makes the 8-OG-containing RNA resistant to nuclease degradation [116]. However, it has been proposed that these proteins may be able to recognize and distinguish the oxidized RNA molecule from normal ones, their binding thus contributing to the fidelity of translation in cells by sequestering the damaged RNA from the translational machinery [116–118].

The human PNP protein binds preferentially to 8-OG-containing RNA and cellular amounts of human PNP protein decrease rapidly by exposure to agents inducing oxidative stress, while amounts of other proteins in the cells do not change after these treatments [118]. Recently, human YB-1 was demonstrated to be a component of P-bodies where active degradation of mRNA occurs. YB-1 is translocated from P-bodies to stress granules during oxidative stress, which suggests a dynamic link between P-bodies and stress granules under oxidative stress [119].

It is possible that RNA quality control mechanisms are defective or inefficient in cancer cells as well as cells of neurodegenerative diseases. Further elucidation of the mechanisms of repair or avoidance of RNA damage and their potential role in preventing human diseases might provide new approaches to therapy in a number of conditions that are so far untreatable.

## 9. Conclusions

Involvement of RNA oxidation in the process of neurodegeneration has been demonstrated in vulnerable neuronal population in neurodegenerative diseases such as AD and PD as well as in several cellular and animal models of neurodegeneration. Particular emphasis should be placed on the early-stage involvement of RNA oxidation in the process of neurodegeneration, which suggests a primary role of RNA oxidation in the disease process. Indeed, oxidized RNA is associated with a disturbance in protein synthesis *in vitro* and *in vivo*. There are presently only a small number of studies suggesting the existence of coping mechanisms for RNA damage. The known mechanisms and the implications of their failure may be only the “tip of iceberg” of the role of sublethal RNA oxidation in chronic disease. Understanding the consequences and cellular handling mechanisms of oxidative RNA damage may provide clues to the pathophysiology of neurodegenerative diseases and form the basis for better therapeutic strategies.

## Figures and Tables

**Figure 1.**
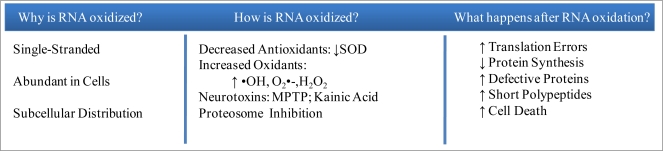
RNA Susceptibility to Oxidation is Sublethal Resulting in Chronic Neurodegeneration.

## References

[1] Hirtz D, Thurman DJ, Gwinn-Hardy K, Mohamed M, Chaudhuri AR, Zalutsky R (2007). How common are the “common” neurologic disorders?. Neurology.

[2] Sayre LM, Smith MA, Perry G (2001). Chemistry and biochemistry of oxidative stress in neurodegenerative disease. Curr. Med. Chem..

[3] Ischiropoulos H, Beckman JS (2003). Oxidative stress and nitration in neurodegeneration: cause, effect, or association?. J. Clin. Invest..

[4] Jenner P (2003). Oxidative stress in Parkinson's disease. Ann. Neurol..

[5] Andersen JK (2004). Oxidative stress in neurodegeneration: cause or consequence?. Nat. Med..

[6] Barnham KJ, Masters CL, Bush AI (2004). Neurodegenerative diseases and oxidative stress. Nat Rev Drug Discov.

[7] Barber SC, Mead RJ, Shaw PJ (2006). Oxidative stress in ALS: a mechanism of neurodegeneration and a therapeutic target. Biochim. Biophys. Acta.

[8] Lin MT, Beal MF (2006). Mitochondrial dysfunction and oxidative stress in neurodegenerative diseases. Nature.

[9] Nunomura A, Castellani RJ, Zhu X, Moreira PI, Perry G, Smith MA (2006). Involvement of oxidative stress in Alzheimer disease. J. Neuropathol. Exp. Neurol..

[10] Nunomura A, Perry G, Pappolla MA, Wade R, Hirai K, Chiba S, Smith MA (1999). RNA oxidation is a prominent feature of vulnerable neurons in Alzheimer's disease. J. Neurosci..

[11] Li Z, Wu J, Deleo CJ (2006). RNA damage and surveillance under oxidative stress. IUBMB life.

[12] Bregeon D, Sarasin A (2005). Hypothetical role of RNA damage avoidance in preventing human disease. Mutat. Res..

[13] Moreira PI, Nunomura A, Nakamura M, Takeda A, Shenk JC, Aliev G, Smith MA, Perry G (2008). Nucleic acid oxidation in Alzheimer disease. Free Radic. Biol. Med..

[14] Hofer T, Seo AY, Prudencio M, Leeuwenburgh C (2006). A method to determine RNA and DNA oxidation simultaneously by HPLC-ECD: greater RNA than DNA oxidation in rat liver after doxorubicin administration. Biol. Chem..

[15] Shen Z, Wu W, Hazen SL (2000). Activated leukocytes oxidatively damage DNA, RNA, and the nucleotide pool through halide-dependent formation of hydroxyl radical. Biochemistry (Mosc)..

[16] Wamer WG, Wei RR (1997). *In vitro* photooxidation of nucleic acids by ultraviolet A radiation. Photochem. Photobiol..

[17] Hofer T, Badouard C, Bajak E, Ravanat JL, Mattsson A, Cotgreave IA (2005). Hydrogen peroxide causes greater oxidation in cellular RNA than in DNA. Biol. Chem..

[18] Fiala ES, Conaway CC, Mathis JE (1989). Oxidative DNA and RNA damage in the livers of Sprague-Dawley rats treated with the hepatocarcinogen 2-nitropropane. Cancer Res..

[19] Weimann A, Belling D, Poulsen HE (2002). Quantification of 8-oxo-guanine and guanine as the nucleobase, nucleoside and deoxynucleoside forms in human urine by high-performance liquid chromatography-electrospray tandem mass spectrometry. Nucleic Acids Res.

[20] Szymanski M, Barciszewska MZ, Erdmann VA, Barciszewski J (2005). A new frontier for molecular medicine: noncoding RNAs. Biochim. Biophys. Acta.

[21] Costa FF (2005). Non-coding RNAs: new players in eukaryotic biology. Gene.

[22] Cao X, Yeo G, Muotri AR, Kuwabara T, Gage FH (2006). Noncoding RNAs in the mammalian central nervous system. Annu. Rev. Neurosci..

[23] Mehler MF, Mattick JS (2006). Non-coding RNAs in the nervous system. J. Physiol..

[24] Taft RJ, Pheasant M, Mattick JS (2007). The relationship between non-protein-coding DNA and eukaryotic complexity. Bioessays.

[25] Perkins DO, Jeffries C, Sullivan P (2005). Expanding the ‘central dogma’: the regulatory role of nonprotein coding genes and implications for the genetic liability to schizophrenia. Mol. Psychiatry.

[26] Taylor JP, Hardy J, Fischbeck KH (2002). Toxic proteins in neurodegenerative disease. Science.

[27] van Leeuwen FW, de Kleijn DP, van den Hurk HH, Neubauer A, Sonnemans MA, Sluijs JA, Koycu S, Ramdjielal RD, Salehi A, Martens GJ, Grosveld FG, Peter J, Burbach H, Hol EM (1998). Frameshift mutants of beta amyloid precursor protein and ubiquitin-B in Alzheimer's and Down patients. Science.

[28] Lee JW, Beebe K, Nangle LA, Jang J, Longo-Guess CM, Cook SA, Davisson MT, Sundberg JP, Schimmel P, Ackerman SL (2006). Editing-defective tRNA synthetase causes protein misfolding and neurodegeneration. Nature.

[29] Evans MD, Dizdaroglu M, Cooke MS (2004). Oxidative DNA damage and disease: induction, repair and significance. Mutat. Res..

[30] Cooke MS, Olinski R, Evans MD (2006). Does measurement of oxidative damage to DNA have clinical significance?. Clin. Chim. Acta.

[31] Kasai H, Crain PF, Kuchino Y, Nishimura S, Ootsuyama A, Tanooka H (1986). Formation of 8-hydroxyguanine moiety in cellular DNA by agents producing oxygen radicals and evidence for its repair. Carcinogenesis.

[32] Ames BN, Gold LS (1991). Endogenous mutagens and the causes of aging and cancer. Mutat. Res..

[33] Yanagawa H, Ogawa Y, Ueno M (1992). Redox ribonucleosides. Isolation and characterization of 5-hydroxyuridine, 8-hydroxyguanosine, and 8-hydroxyadenosine from Torula yeast RNA. J. Biol. Chem..

[34] Schneider JE, Phillips JR, Pye Q, Maidt ML, Price S, Floyd RA (1993). Methylene blue and rose bengal photoinactivation of RNA bacteriophages: comparative studies of 8-oxoguanine formation in isolated RNA. Arch. Biochem. Biophys..

[35] Rhee Y, Valentine MR, Termini J (1995). Oxidative base damage in RNA detected by reverse transcriptase. Nucleic Acids Res.

[36] Barciszewski J, Barciszewska MZ, Siboska G, Rattan SI, Clark BF (1999). Some unusual nucleic acid bases are products of hydroxyl radical oxidation of DNA and RNA. Mol. Biol. Rep..

[37] Yin B, Whyatt RM, Perera FP, Randall MC, Cooper TB, Santella RM (1995). Determination of 8-hydroxydeoxyguanosine by an immunoaffinity chromatography-monoclonal antibody-based ELISA. Free Radic. Biol. Med..

[38] Park EM, Shigenaga MK, Degan P, Korn TS, Kitzler JW, Wehr CM, Kolachana P, Ames BN (1992). Assay of excised oxidative DNA lesions: isolation of 8-oxoguanine and its nucleoside derivatives from biological fluids with a monoclonal antibody column. Proc. Natl. Acad. Sci. U. S. A..

[39] Zhang J, Perry G, Smith MA, Robertson D, Olson SJ, Graham DG, Montine TJ (1999). Parkinson's disease is associated with oxidative damage to cytoplasmic DNA and RNA in substantia nigra neurons. Am. J. Pathol..

[40] Nunomura A, Perry G, Aliev G, Hirai K, Takeda A, Balraj EK, Jones PK, Ghanbari H, Wataya T, Shimohama S, Chiba S, Atwood CS, Petersen RB, Smith MA (2001). Oxidative damage is the earliest event in Alzheimer disease. J. Neuropathol. Exp. Neurol..

[41] Nunomura A, Perry G, Pappolla MA, Friedland RP, Hirai K, Chiba S, Smith MA (2000). Neuronal oxidative stress precedes amyloid-beta deposition in Down syndrome. J. Neuropathol. Exp. Neurol..

[42] Nunomura A, Chiba S, Kosaka K, Takeda A, Castellani RJ, Smith MA, Perry G (2002). Neuronal RNA oxidation is a prominent feature of dementia with Lewy bodies. Neuroreport.

[43] Guentchev M, Siedlak SL, Jarius C, Tagliavini F, Castellani RJ, Perry G, Smith MA, Budka H (2002). Oxidative damage to nucleic acids in human prion disease. Neurobiol. Dis..

[44] Hayashi M, Arai N, Satoh J, Suzuki H, Katayama K, Tamagawa K, Morimatsu Y (2002). Neurodegenerative mechanisms in subacute sclerosing panencephalitis. J. Child Neurol..

[45] Nunomura A, Chiba S, Lippa CF, Cras P, Kalaria RN, Takeda A, Honda K, Smith MA, Perry G (2004). Neuronal RNA oxidation is a prominent feature of familial Alzheimer's disease. Neurobiol. Dis..

[46] Petersen RB, Siedlak SL, Lee HG, Kim YS, Nunomura A, Tagliavini F, Ghetti B, Cras P, Moreira PI, Castellani RJ, Guentchev M, Budka H, Ironside JW, Gambetti P, Smith MA, Perry G (2005). Redox metals and oxidative abnormalities in human prion diseases. Acta Neuropathol.

[47] Hayashi M, Araki S, Kohyama J, Shioda K, Fukatsu R (2005). Oxidative nucleotide damage and superoxide dismutase expression in the brains of xeroderma pigmentosum group A and Cockayne syndrome. Brain Dev..

[48] Tateyama M, Takeda A, Onodera Y, Matsuzaki M, Hasegawa T, Nunomura A, Hirai K, Perry G, Smith MA, Itoyama Y (2003). Oxidative stress and predominant Abeta42(43) deposition in myopathies with rimmed vacuoles. Acta Neuropathol. (Berl)..

[49] Martinet W, de Meyer GR, Herman AG, Kockx MM (2004). Reactive oxygen species induce RNA damage in human atherosclerosis. Eur. J. Clin. Invest..

[50] Hofer T, Marzetti E, Xu J, Seo AY, Gulec S, Knutson MD, Leeuwenburgh C, Dupont-Versteegden EE (2008). Increased iron content and RNA oxidative damage in skeletal muscle with aging and disuse atrophy. Exp. Gerontol..

[51] Shan X, Tashiro H, Lin CL (2003). The identification and characterization of oxidized RNAs in Alzheimer's disease. J. Neurosci..

[52] Honda K, Smith MA, Zhu X, Baus D, Merrick WC, Tartakoff AM, Hattier T, Harris PL, Siedlak SL, Fujioka H, Liu Q, Moreira PI, Miller FP, Nunomura A, Shimohama S, Perry G (2005). Ribosomal RNA in Alzheimer disease is oxidized by bound redox-active iron. J. Biol. Chem..

[53] Ding Q, Markesbery WR, Chen Q, Li F, Keller JN (2005). Ribosome dysfunction is an early event in Alzheimer's disease. J. Neurosci..

[54] Ding Q, Markesbery WR, Cecarini V, Keller JN (2006). Decreased RNA, and increased RNA oxidation, in ribosomes from early Alzheimer's disease. Neurochem. Res..

[55] Shan X, Lin CL (2006). Quantification of oxidized RNAs in Alzheimer's disease. Neurobiol. Aging.

[56] Abe T, Tohgi H, Isobe C, Murata T, Sato C (2002). Remarkable increase in the concentration of 8-hydroxyguanosine in cerebrospinal fluid from patients with Alzheimer's disease. J. Neurosci. Res..

[57] Kikuchi A, Takeda A, Onodera H, Kimpara T, Hisanaga K, Sato N, Nunomura A, Castellani RJ, Perry G, Smith MA, Itoyama Y (2002). Systemic increase of oxidative nucleic acid damage in Parkinson's disease and multiple system atrophy. Neurobiol. Dis..

[58] Abe T, Isobe C, Murata T, Sato C, Tohgi H (2003). Alteration of 8-hydroxyguanosine concentrations in the cerebrospinal fluid and serum from patients with Parkinson's disease. Neurosci. Lett..

[59] Liu J, Head E, Gharib AM, Yuan W, Ingersoll RT, Hagen TM, Cotman CW, Ames BN (2002). Memory loss in old rats is associated with brain mitochondrial decay and RNA/DNA oxidation: partial reversal by feeding acetyl-L-carnitine and/or R-alpha -lipoic acid. Proc. Natl. Acad. Sci. U. S. A..

[60] Row BW, Liu R, Xu W, Kheirandish L, Gozal D (2003). Intermittent hypoxia is associated with oxidative stress and spatial learning deficits in the rat. Am. J. Respir. Crit. Care Med..

[61] Yamaguchi H, Kajitani K, Dan Y, Furuichi M, Ohno M, Sakumi K, Kang D, Nakabeppu Y (2006). MTH1, an oxidized purine nucleoside triphosphatase, protects the dopamine neurons from oxidative damage in nucleic acids caused by 1-methyl-4-phenyl-1,2,3,6-tetrahydropyridine. Cell Death Differ..

[62] Foksinski M, Rozalski R, Guz J, Ruszkowska B, Sztukowska P, Piwowarski M, Klungland A, Olinski R (2004). Urinary excretion of DNA repair products correlates with metabolic rates as well as with maximum life spans of different mammalian species. Free Radic. Biol. Med..

[63] Javitch JA, D'Amato RJ, Strittmatter SM, Snyder SH (1985). Parkinsonism-inducing neurotoxin, N-methyl-4-phenyl-1,2,3,6 -tetrahydropyridine: uptake of the metabolite N-methyl-4-phenylpyridine by dopamine neurons explains selective toxicity. Proc. Natl. Acad. Sci. U. S. A..

[64] Wang Q, Yu S, Simonyi A, Sun GY, Sun AY (2005). Kainic acid-mediated excitotoxicity as a model for neurodegeneration. Mol. Neurobiol..

[65] Kajitani K, Yamaguchi H, Dan Y, Furuichi M, Kang D, Nakabeppu Y (2006). MTH1, an oxidized purine nucleoside triphosphatase, suppresses the accumulation of oxidative damage of nucleic acids in the hippocampal microglia during kainate-induced excitotoxicity. J. Neurosci..

[66] Rosen DR, Siddique T, Patterson D, Figlewicz DA, Sapp P, Hentati A, Donaldson D, Goto J, O'Regan JP, Deng HX (1993). Mutations in Cu/Zn superoxide dismutase gene are associated with familial amyotrophic lateral sclerosis. Nature.

[67] Gurney ME, Pu H, Chiu AY, Dal Canto MC, Polchow CY, Alexander DD, Caliendo J, Hentati A, Kwon YW, Deng HX (1994). Motor neuron degeneration in mice that express a human Cu, Zn superoxide dismutase mutation. Science.

[68] Chang Y, Shan X, Lin CL (2004). RNA oxidation is an early event preceding motor neuron death in ALS. Soc. Neurosci. Abstr..

[69] Ding Q, Dimayuga E, Markesbery WR, Keller JN (2004). Proteasome inhibition increases DNA and RNA oxidation in astrocyte and neuron cultures. J. Neurochem..

[70] Shan X, Chang Y, Lin CL (2007). Messenger RNA oxidation is an early event preceding cell death and causes reduced protein expression. FASEB J..

[71] Ding Q, Cecarini V, Keller JN (2007). Interplay between protein synthesis and degradation in the CNS: physiological and pathological implications. Trends Neurosci..

[72] Keller JN, Schmitt FA, Scheff SW, Ding Q, Chen Q, Butterfield DA, Markesbery WR (2005). Evidence of increased oxidative damage in subjects with mild cognitive impairment. Neurology.

[73] Butterfield DA, Reed TT, Perluigi M, De Marco C, Coccia R, Keller JN, Markesbery WR, Sultana R (2007). Elevated levels of 3-nitrotyrosine in brain from subjects with amnestic mild cognitive impairment: implications for the role of nitration in the progression of Alzheimer's disease. Brain Res..

[74] Pratico D, Clark CM, Liun F, Rokach J, Lee VY, Trojanowski JQ (2002). Increase of brain oxidative stress in mild cognitive impairment: a possible predictor of Alzheimer disease. Arch. Neurol..

[75] Migliore L, Fontana I, Trippi F, Colognato R, Coppede F, Tognoni G, Nucciarone B, Siciliano G (2005). Oxidative DNA damage in peripheral leukocytes of mild cognitive impairment and AD patients. Neurobiol. Aging.

[76] Rinaldi P, Polidori MC, Metastasio A, Mariani E, Mattioli P, Cherubini A, Catani M, Cecchetti R, Senin U, Mecocci P (2003). Plasma antioxidants are similarly depleted in mild cognitive impairment and in Alzheimer's disease. Neurobiol. Aging.

[77] Guidi I, Galimberti D, Lonati S, Novembrino C, Bamonti F, Tiriticco M, Fenoglio C, Venturelli E, Baron P, Bresolin N, Scarpini E (2006). Oxidative imbalance in patients with mild cognitive impairment and Alzheimer's disease. Neurobiol. Aging.

[78] Moreira PI, Zhu X, Nunomura A, Smith MA, Perry G (2006). Therapeutic options in Alzheimer's disease. Expert Rev. Neurother..

[79] Liu Q, Xie F, Rolston R, Moreira PI, Nunomura A, Zhu X, Smith MA, Perry G (2007). Prevention and treatment of Alzheimer disease and aging: antioxidants. Mini Rev. Med. Chem..

[80] Sayre LM, Perry G, Smith MA (1999). *In situ* methods for detection and localization of markers of oxidative stress: application in neurodegenerative disorders. Methods Enzymol..

[81] Smith MA, Perry G, Richey PL, Sayre LM, Anderson VE, Beal MF, Kowall N (1996). Oxidative damage in Alzheimer's. Nature.

[82] Sayre LM, Zelasko DA, Harris PL, Perry G, Salomon RG, Smith MA (1997). 4-Hydroxynonenal-derived advanced lipid peroxidation end products are increased in Alzheimer's disease. J. Neurochem..

[83] Casadesus G, Smith MA, Basu S, Hua J, Capobianco DE, Siedlak SL, Zhu X, Perry G (2007). Increased isoprostane and prostaglandin are prominent in neurons in Alzheimer disease. Mol. Neurodegener..

[84] Castellani RJ, Harris PL, Sayre LM, Fujii J, Taniguchi N, Vitek MP, Founds H, Atwood CS, Perry G, Smith MA (2001). Active glycation in neurofibrillary pathology of Alzheimer disease: N(epsilon)-(carboxymethyl) lysine and hexitol-lysine. Free Radic. Biol. Med..

[85] Coyle JT, Puttfarcken P (1993). Oxidative stress, glutamate, and neurodegenerative disorders. Science.

[86] Mattson MP, Chan SL, Duan W (2002). Modification of brain aging and neurodegenerative disorders by genes, diet, and behavior. Physiol. Rev..

[87] Halliwell B (1992). Reactive oxygen species and the central nervous system. J. Neurochem..

[88] Joenje H (1989). Genetic toxicology of oxygen. Mutat. Res..

[89] Takahashi MA, Asada K (1983). Superoxide anion permeability of phospholipid membranes and chloroplast thylakoids. Arch. Biochem. Biophys..

[90] Schubert J, Wilmer JW (1991). Does hydrogen peroxide exist "free" in biological systems?. Free Radic. Biol. Med..

[91] Hirai K, Aliev G, Nunomura A, Fujioka H, Russell RL, Atwood CS, Johnson AB, Kress Y, Vinters HV, Tabaton M, Shimohama S, Cash AD, Siedlak SL, Harris PL, Jones PK, Petersen RB, Perry G, Smith MA (2001). Mitochondrial abnormalities in Alzheimer's disease. J. Neurosci..

[92] Perry G, Nunomura A, Cash AD, Taddeo MA, Hirai K, Aliev G, Avila J, Wataya T, Shimohama S, Atwood CS, Smith MA (2002). Reactive oxygen: its sources and significance in Alzheimer disease. J. Neural Transm. Suppl..

[93] Perry G, Nunomura A, Hirai K, Zhu X, Perez M, Avila J, Castellani RJ, Atwood CS, Aliev G, Sayre LM, Takeda A, Smith MA (2002). Is oxidative damage the fundamental pathogenic mechanism of Alzheimer's and other neurodegenerative diseases?. Free Radic. Biol. Med..

[94] Smith MA, Nunomura A, Zhu X, Takeda A, Perry G (2000). Metabolic, metallic, and mitotic sources of oxidative stress in Alzheimer disease. Antioxid. Redox Signal..

[95] Gu G, Reyes PE, Golden GT, Woltjer RL, Hulette C, Montine TJ, Zhang J (2002). Mitochondrial DNA deletions/rearrangements in parkinson disease and related neurodegenerative disorders. J. Neuropathol. Exp. Neurol..

[96] Schapira AH, Cooper JM, Dexter D, Clark JB, Jenner P, Marsden CD (1990). Mitochondrial complex I deficiency in Parkinson's disease. J. Neurochem..

[97] Sofic E, Riederer P, Heinsen H, Beckmann H, Reynolds GP, Hebenstreit G, Youdim MB (1988). Increased iron (III) and total iron content in post mortem substantia nigra of parkinsonian brain. J. Neural Transm..

[98] Berg D, Roggendorf W, Schroder U, Klein R, Tatschner T, Benz P, Tucha O, Preier M, Lange KW, Reiners K, Gerlach M, Becker G (2002). Echogenicity of the substantia nigra: association with increased iron content and marker for susceptibility to nigrostriatal injury. Arch. Neurol..

[99] Ishibashi T, Hayakawa H, Ito R, Miyazawa M, Yamagata Y, Sekiguchi M (2005). Mammalian enzymes for preventing transcriptional errors caused by oxidative damage. Nucleic Acids Res.

[100] Taddei F, Hayakawa H, Bouton M, Cirinesi A, Matic I, Sekiguchi M, Radman M (1997). Counteraction by MutT protein of transcriptional errors caused by oxidative damage. Science.

[101] Tanaka M, Chock PB, Stadtman ER (2007). Oxidized messenger RNA induces translation errors. Proc. Natl. Acad. Sci. U. S. A..

[102] Gong X, Tao R, Li Z (2006). Quantification of RNA damage by reverse transcription polymerase chain reactions. Anal. Biochem..

[103] Bellacosa A, Moss EG (2003). RNA repair: damage control. Curr. Biol..

[104] Deutscher MP (2006). Degradation of RNA in bacteria: comparison of mRNA and stable RNA. Nucleic Acids Res.

[105] Sheth U, Parker R (2003). Decapping and decay of messenger RNA occur in cytoplasmic processing bodies. Science.

[106] Sweet TJ, Boyer B, Hu W, Baker KE, Coller J (2007). Microtubule disruption stimulates P-body formation. RNA.

[107] Kedersha N, Stoecklin G, Ayodele M, Yacono P, Lykke-Andersen J, Fritzler MJ, Scheuner D, Kaufman RJ, Golan DE, Anderson P (2005). Stress granules and processing bodies are dynamically linked sites of mRNP remodeling. J. Cell Biol..

[108] Aas PA, Otterlei M, Falnes PO, Vagbo CB, Skorpen F, Akbari M, Sundheim O, Bjoras M, Slupphaug G, Seeberg E, Krokan HE (2003). Human and bacterial oxidative demethylases repair alkylation damage in both RNA and DNA. Nature.

[109] Krokan HE, Kavli B, Slupphaug G (2004). Novel aspects of macromolecular repair and relationship to human disease. J. Mol. Med..

[110] Nakabeppu Y, Tsuchimoto D, Ichinoe A, Ohno M, Ide Y, Hirano S, Yoshimura D, Tominaga Y, Furuichi M, Sakumi K (2004). Biological significance of the defense mechanisms against oxidative damage in nucleic acids caused by reactive oxygen species: from mitochondria to nuclei. Ann. N. Y. Acad. Sci..

[111] Hayakawa H, Hofer A, Thelander L, Kitajima S, Cai Y, Oshiro S, Yakushiji H, Nakabeppu Y, Kuwano M, Sekiguchi M (1999). Metabolic fate of oxidized guanine ribonucleotides in mammalian cells. Biochemistry (Mosc)..

[112] Nakabeppu Y, Kajitani K, Sakamoto K, Yamaguchi H, Tsuchimoto D (2006). MTH1, an oxidized purine nucleoside triphosphatase, prevents the cytotoxicity and neurotoxicity of oxidized purine nucleotides. DNA Repair (Amst.).

[113] Ito R, Hayakawa H, Sekiguchi M, Ishibashi T (2005). Multiple enzyme activities of Escherichia coli MutT protein for sanitization of DNA and RNA precursor pools. Biochemistry (Mosc)..

[114] Furuta A, Iida T, Nakabeppu Y, Iwaki T (2001). Expression of hMTH1 in the hippocampi of control and Alzheimer's disease. Neuroreport.

[115] Shimura-Miura H, Hattori N, Kang D, Miyako K, Nakabeppu Y, Mizuno Y (1999). Increased 8-oxo-dGTPase in the mitochondria of substantia nigral neurons in Parkinson's disease. Ann. Neurol..

[116] Hayakawa H, Kuwano M, Sekiguchi M (2001). Specific binding of 8-oxoguanine-containing RNA to polynucleotide phosphorylase protein. Biochemistry (Mosc)..

[117] Hayakawa H, Sekiguchi M (2006). Human polynucleotide phosphorylase protein in response to oxidative stress. Biochemistry (Mosc)..

[118] Hayakawa H, Uchiumi T, Fukuda T, Ashizuka M, Kohno K, Kuwano M, Sekiguchi M (2002). Binding capacity of human YB-1 protein for RNA containing 8-oxoguanine. Biochemistry (Mosc)..

[119] Yang WH, Bloch DB (2007). Probing the mRNA processing body using protein macroarrays and “autoantigenomics”. RNA.

